# Glutamate Within the Marmoset Anterior Hippocampus Interacts with Area 25 to Regulate the Behavioral and Cardiovascular Correlates of High-Trait Anxiety

**DOI:** 10.1523/JNEUROSCI.2451-18.2018

**Published:** 2019-04-17

**Authors:** Jorge L. Zeredo, Shaun K.L. Quah, Chloe U. Wallis, Laith Alexander, Gemma J. Cockcroft, Andrea M. Santangelo, Jing Xia, Yoshiro Shiba, Jeffrey W. Dalley, Rudolf N. Cardinal, Angela C. Roberts, Hannah F. Clarke

**Affiliations:** ^1^Graduate Program in Health Science and Technology, University of Brasilia, Brasilia, Brazil,; ^2^Department of Physiology, Development and Neuroscience, University of Cambridge, Cambridge CB2 3DY, United Kingdom,; ^3^Department of Psychology, University of Cambridge, Cambridge, CB2 3EB, United Kingdom,; ^4^Department of Psychiatry, and; ^5^Liaison Psychiatry Service, Cambridge and Peterborough NHS Foundation Trust, Cambridge Biomedical Campus, Cambridg, CB2 OQQ, United Kingdom

**Keywords:** anxiety, area 25, cardiovascular, glutamate, hippocampus, marmoset

## Abstract

High-trait anxiety is a risk factor for the development of affective disorders and has been associated with decreased cardiovascular and behavioral responsivity to acute stressors in humans that may increase the risk of developing cardiovascular disease. Although human neuroimaging studies of high-trait anxiety reveals dysregulation in primate cingulate areas 25 and 32 and the anterior hippocampus (aHipp) and rodent studies reveal the importance of aHipp glutamatergic hypofunction, the causal involvement of aHipp glutamate and its interaction with these areas in the primate brain is unknown. Accordingly, we correlated marmoset trait anxiety scores to their postmortem aHipp glutamate levels and showed that low glutamate in the right aHipp is associated with high-trait anxiety in marmosets. Moreover, pharmacologically increasing aHipp glutamate reduced anxiety levels in highly anxious marmosets in two uncertainty-based tests of anxiety: exposure to a human intruder with uncertain intent and unpredictable loud noise. In the human intruder test, increasing aHipp glutamate decreased anxiety by increasing approach to the intruder. In the unpredictable threat test, animals showed blunted behavioral and cardiovascular responsivity after control infusions, which was normalized by increasing aHipp glutamate. However, this aHipp-mediated anxiolytic effect was blocked by simultaneous pharmacological inactivation of area 25, but not area 32, areas which when inactivated independently reduced and had no effect on anxiety, respectively. These findings provide causal evidence in male and female primates that aHipp glutamatergic hypofunction and its regulation by area 25 contribute to the behavioral and cardiovascular symptoms of endogenous high-trait anxiety.

**SIGNIFICANCE STATEMENT** High-trait anxiety predisposes sufferers to the development of anxiety and depression. Although neuroimaging of these disorders and rodent modeling implicate dysregulation in hippocampal glutamate and the subgenual/perigenual cingulate cortices (areas 25/32), the causal involvement of these structures in endogenous high-trait anxiety and their interaction are unknown. Here, we demonstrate that increased trait anxiety in marmoset monkeys correlates with reduced hippocampal glutamate and that increasing hippocampal glutamate release in high-trait-anxious monkeys normalizes the aberrant behavioral and cardiovascular responsivity to potential threats. This normalization was blocked by simultaneous inactivation of area 25, but not area 32. These findings provide casual evidence in primates that hippocampal glutamatergic hypofunction regulates endogenous high-trait anxiety and the hippocampal–area 25 circuit is a potential therapeutic target.

## Introduction

Trait anxiety refers to stable individual differences in how individuals respond to acutely stressful or threatening situations. High-trait-anxious individuals are predisposed to developing anxiety and depression (Weger and Sandi, 2018), are more likely to interpret ambiguous stimuli as threatening (Mathews et al., 1989), and show reduced motivation (Ginty et al., 2015). Physiologically, increased anxiety is normally associated with increases in the autonomic indices of fear in response to fearful stimuli. However, a principal diagnosis in the domain of “anxiety” covers a wide spectrum of anxiety-related and comorbid disorders and, although enhanced fear physiology is seen in specific phobia patients, there is increasing evidence that high chronic (trait) levels of anxiety and negative affect are associated with blunted cardiovascular responsivity and a failure to show the adaptive cardiovascular changes that characterize a healthy “fight-or-flight” response (Ginty and Conklin, 2011; Brindle et al., 2013; Lang et al., 2014, 2016; Souza et al., 2015). Moreover, the cardiovascular function of high-trait-anxious and negative individuals does not discriminate between acutely stressful and nonstressful conditions (Carroll et al., 2017), an adaptive failure that may contribute to the increased incidence of cardiovascular disease in patients suffering from depression and anxiety (Stapelberg et al., 2011, 2012).

Numerous models of high-trait anxiety have been developed to investigate the neurobiology of these disorders. One such model of rodent prenatal stress links a high-trait/depressive phenotype in the offspring with decreased depolarization-evoked glutamate release in the ventral hippocampus (vHipp) (Marrocco et al., 2012, 2014). Although the relevance of such models to endogenous high-trait anxiety is uncertain, early life stress is a known risk factor for affective disorders and there is growing evidence that reduced hippocampal glutamatergic function contributes to affective disorder pathophysiology. Certainly, hippocampal glutamatergic hypofunction correlates with depressive illness duration (Block et al., 2009; de Diego-Adeliño et al., 2013) and disrupted glutamatergic signaling pathways are associated with anxious/depressive behaviors in mice and humans (Tordera et al., 2007; Garcia-Garcia et al., 2009; Duric et al., 2013). However, these findings conflict with studies of nonanxious rodents in which reductions in vHipp glutamate function are anxiolytic in ethological tests of anxiety (Barkus et al., 2010) and the anxiolytic effects of hippocampal lesions in nonanxious primates (Chudasama et al., 2008, 2009). Therefore, there may be fundamental differences in how hippocampal glutamate contributes to healthy and high-trait-anxious/affective disorder states. The hippocampus modulates such behavior, in part, via a network of medial prefrontal cortex (mPFC) brain regions including Brodmann's area 25 and area 32 and changes in anterior hippocampal (aHipp, the primate equivalent of the vHipp) connectivity with the mPFC are implicated in affective disorders in humans (Dickie et al., 2011; Hamilton et al., 2011; Treadway et al., 2015). Rodent studies also highlight the importance of vHipp–mPFC connectivity in the control of anxiety-related behavior (Adhikari et al., 2010; Padilla-Coreano et al., 2016). However, the contributions of hippocampal glutamate and hippocampal–mPFC circuitry in the behavioral and cardiovascular symptoms of endogenous high-trait anxiety remain unknown.

We have recently demonstrated the utility of a marmoset model for probing the behavioral and cardiovascular correlates of mPFC function and conditioned fear (Wallis et al., 2017) and described a subpopulation of marmosets with an endogenous high-trait anxiety phenotype that is associated with impaired discriminative Pavlovian conditioning, blunted amygdala serotonin function, and reduced dorsal anterior cingulate cortex volume (Shiba et al., 2014; Mikheenko et al., 2015). However, the contribution of hippocampal glutamate to this phenotype is unknown. Therefore, the first aim of the present study (Experiment 1) was to assess whether reduced postmortem hippocampal glutamate levels predicted heightened responsivity of a marmoset cohort to an unknown human. Having established a negative correlation between hippocampal glutamate and such responsivity, the second aim (Experiment 2) was to determine whether hippocampal glutamate and high-trait anxiety were causally related. Therefore, we used anatomically specific intracerebral infusions to increase presynaptic hippocampal glutamate release in high-trait-anxious monkeys and assessed its effectiveness in ameliorating the behavioral and cardiovascular correlates of negative emotion in two anxiety tests, the human intruder paradigm, which measures uncertainty-based behavior in the home cage, and an unpredictable threat test presented in an automated test apparatus, which measures real-time behavioral and cardiovascular responses. We then determined whether this amelioration used hippocampal–mPFC circuitry.

## Materials and Methods

### Animals

All of the marmosets (*Callithrix jacchus*) used in these studies were bred onsite at the University of Cambridge Marmoset Breeding Colony and were housed in male/female pairs (males were vasectomized). They were kept in a 12 h light/dark cycle (lights on at 7:00 A.M., lights off at 7:00 P.M.) in a controlled environment of 22 ± 1°C and 50 ± 1% humidity. Their cages contained a variety of environmental enrichment aids including boxes to play in and suspended ladders, wooden branches, and ropes to climb and swing on. Animals were fed a varied diet including fruit, rusk, malt loaf, peanuts, eggs, sandwiches, and weekend treats and had *ad libitum* access to water. All procedures were performed in accordance with the UK Animals (Scientific Procedures) Act of 1986 and the University of Cambridge Animal Welfare and Ethical Review Board.

### Experiment 1

#### Hippocampal neurochemical quantification

To assess the contribution of hippocampal neurochemistry to the high-trait-anxious phenotype, stored hippocampal tissue from 12 marmosets (six males, six females) with a range of anxiety scores on the human intruder test (described below) was analyzed. Behavioral and cardiovascular data from these marmosets have previously been described previously (Shiba et al., 2014). After euthanasia, the brain was removed and the anterior two-thirds of the left and right hippocampus was visualized, immediately dissected out, and flash frozen into liquid nitrogen before storage at −80°C. For analysis, these tissue samples were thawed on ice and homogenized with 500 μl of Lysis Buffer NL from the Qiagen Qproteome Nuclear Protein Kit before 250 μl of this sample was analyzed using reverse-phase high-performance liquid chromatography (HPLC) and electrochemical detection for both monoamines and glutamate as described previously (Tsai et al., 1997; Clarke et al., 2004). Briefly, monoamines were separated on a C18 silica-based analytical column (10 × 4.6 mm ODS3; Hypersil, Phenomoex) using a mobile phase consisting of 13.6 g/L KH_2_PO_4_^.^H_2_O, 185 mg/L octane sulfonic acid, and 18% methanol, pH 2.75, delivered at 0.8 ml/min by a dual piston pump. Detection was achieved electrochemically using a dual electrode analytical cell and electrochemical detector with electrode 1 set at −150 mV and electrode 2 set at 180 mV with reference to a palladium reference electrode. For the measurement of glutamate, 6 μl of each sample was reacted with an equal volume of a derivatizing agent containing o-phthalaldehyde and β-mercaptoethanol for 2 min at room temperature. The derivatizing solution was prepared daily by diluting 1 ml of stock solution containing 27.5 mg o-phthalaldehyde, 1 ml of methanol, 5 μl of β-mercaptoethanol, and 9 ml of 0.4 m potassium tetraborate buffer, pH 10.4, in 3 ml of potassium tetraborate buffer. Glutamate was detected by HPLC and fluorescence detection (CMA 280) with excitation and emission wavelengths of 315–370 nm and 395–545 nm, respectively. Separation was achieved at 24°C using a Hypersil ODS 5 μm analytical column (80 × 4.6 mm; HPLC Technology) and a mobile phase consisting of 100 mm Na_2_HPO_4_ and 30% methanol, adjusted to pH 6.38 with orthophosphoric acid, which was delivered at 1 ml/min. Before use, the buffer was filtered through a 0.2 μm filter under vacuum. The HPLC system was calibrated using standards containing known amounts of monoamines and glutamate. Data were acquired online and the signals were integrated using Chromeleon software version 6.2 (Dionex). The resulting monoamine and glutamate levels of each animal were then compared with their anxiety scores.

### Experiment 2

To determine whether increases in hippocampal glutamate activity could ameliorate the behavioral and cardiovascular responses associated with high-trait anxiety, we performed two behavioral experiments on marmosets that had been characterized as high anxious (described below). In Experiment 2a, we investigated the effects of selectively increasing aHipp glutamate levels on behavioral performance in the human intruder test in six animals (three males, three females). In Experiment 2b, in six animals (four of which also contributed to Experiment 2a; two females, four males), we investigated the behavioral and cardiovascular effects of selectively increasing aHipp glutamate levels in an unpredictable threat test and also investigated the contribution of mPFC regions 25 and 32 to these effects (Table 1).

**Table 1.** Summary of subjects in behavioral studies of Experiment 2

	1	2	3	4	5	6	7	8
Cannulae targets	aHipp/25/32	aHipp/25/32	aHipp	aHipp/25/32	aHipp/25/32	aHipp	aHipp/25/32	aHipp/25/32
Human intruder test (Experiment 2a)								
Saline 1	✓	✓	✓	✓	✓	✓		
aHipp LY/CGP	✓	✓	✓	✓	✓	✓		
Saline 2	✓	✓	✓	✓	✓	✓		
Unpredictable threat test (Experiment 2b)								
aHipp saline	✓ (2)	✓ (4)		✓ (1)	✓ (2)		✓ (1)	✓ (1)
aHipp LY/CGP	✓ (4)	✓ (5)		✓ (2)	✓ (1)		✓ (3)	
aHipp LY/CGP & musbac 25	✓ (1)	✓ (1)		✓ (3)				✓ (2)
aHipp LY/CGP & musbac 32	✓ (6)	✓ (3)		✓ (4)			✓ (4)	
musbac 25	✓ (3)	✓ (6)		✓ (5)				✓ (3)
musbac 32	✓ (5)	✓ (2)		✓ (6)			✓ (2)	✓ (4)
No. of infusions								
aHipp	10	10	5	10	10	6	14	4
Area 25	3	4	NA	3	3	NA	8	3
Area 32	6	3	NA	2	4	NA	6	1

Checkmarks indicate that the subject took part in that phase of the study. For Experiment 2a, the numbers in parentheses indicate the order in which the infusions were received. Subjects 3 and 6 only had cannulae targeting the aHipp because they underwent prefrontal microdialysis (not reported here). Postmortem histology for subject 5 revealed that the cannulae were not located in areas 25 or 32 and histology for subject 7 revealed that the cannulae were not located in area 25; therefore, their data for these regions are excluded. However, the overall number of infusions that they received in these areas is included for completeness (including other drug combinations that are not reported here).

To allow the selective anatomical manipulation of either the aHipp and/or areas 25 and 32, all 10 animals in Experiment 2 underwent an aseptic surgical procedure to implant intracerebral cannulae targeting either the aHipp alone or the aHipp and areas 25 and 32. Of these, the six animals who also undertook Experiment 2B had another surgery to implant a telemetric blood pressure (BP) monitor into the descending aorta. Both surgeries were completed before the animals began any behavioral testing.

#### Cannulation surgery

Marmosets were premedicated with ketamine hydrochloride (Vetalar; 0.05 ml of a 100 mg solution, i.m.; GE Healthcare and Upjohn) before being given a long-lasting, nonsteroidal, anti-inflammatory analgesic (Carprieve; 0.03 ml of 50 mg/ml carprofen, s.c.; Pfizer,). The animals were intubated and maintained on 2.0–2.5% isoflurane in 0.3 L/min O_2_ and placed into a stereotaxic frame modified for the marmoset (David Kopf Instruments). Pulse-rate, O_2_ saturation, breathing rate, and CO_2_ saturation were all monitored by pulse oximetry and capnography (Microcap Handheld Capnograph; Oridion Capnography) and core body temperature was monitored by a rectal thermometer (TES-1319 K-type digital thermometer; TES Electrical Electronic). Cannulae (Plastics One) were implanted into area 25 [double 7-mm-long cannulae, 1 mm apart, anteroposterior (AP) + 14, lateromedial (LM ± 0.5), area 32 (double 2 mm long cannulae, 1 mm apart; AP +17; LM ± 0.5 at a 30° AP angle), and the aHipp (double 15-mm-long cannula, 1 mm apart, AP + 6, LM ± 5.75/7.75, ventral + 5) with coordinates adjusted where necessary *in situ* according to cortical depth (Roberts et al., 2007). Postoperatively, and when fully recovered, all monkeys were returned to their home cage and then received the analgesic meloxicam (0.1 ml of a 1.5 mg/ml oral suspension; Boehringer Ingelheim) for 3 d, after which they had at least a further 10 d recovery. Cannulae were cleaned every week (and caps and cannula blockers changed) to ensure that the cannula site remained free from infection.

#### Telemetry surgery

Animals were anesthetized as before, the descending aorta was visualized within the abdominal cavity, and the probe catheter of a telemetric BP transmitter (Data Sciences International) was implanted into the aorta as described previously (Braesicke et al., 2005). All monkeys received meloxicam as before in addition to prophylactic treatment with amoxicillin and clavulanic acid (Synulox; 50 mg/ml solution; Pfizer,) for 1 d before and 6 d after telemetry surgery.

#### Drug infusions

Approximately once a week before behavioral testing, animals received infusions of drug or vehicle down the cannulae. The use of chronically implanted cannulae and acute infusions allowed animals to act as their own controls and reduced experimental variation caused by intersubject differences. For all sterile central infusions, the marmoset was held gently in a researcher's hand, the caps and cannula blockers were removed from the guide, and the site was cleaned with 70% alcohol. A sterile injector (Plastics One) connected to a 2 μl gastight syringe in a syringe pump was inserted into the guide cannula for a 2 min drug infusion. Following the infusion, the injector was left in place for a further minute to allow the drug to diffuse before injector removal. Sterile cannula blockers and caps were replaced and the marmoset was returned to its home cage for the relevant pretreatment time. To increase glutamate in the hippocampus, 1 μl of a mixture of the mGlu2/3 receptor antagonist LY341495 (1 ng/μl; Tocris Bioscience) and the GABA_B_ receptor antagonist CGP52432 (1 ng/μl; Tocris Bioscience), the LY/CGP cocktail, was bilaterally infused at a rate of 0.5 μl/min with a 15 min pretreatment time (Marrocco et al., 2012). These receptors both act to limit presynaptic glutamate release and their antagonism therefore acts to increase the amount of glutamate released. To inactivate areas 25 or 32, we used the GABA_A_ agonist muscimol and the GABA_B_ agonist baclofen (0.5 μl of 0.1 mm muscimol/1.0 mm baclofen, referred to here as “musbac”; both from Sigma-Aldrich) infusion at a rate of 0.25 μl/min and a pretreatment time of 25 min. Saline infusions acted as vehicle controls.

#### Human intruder test

##### Defining a high-anxiety trait.

**Figure 1.**
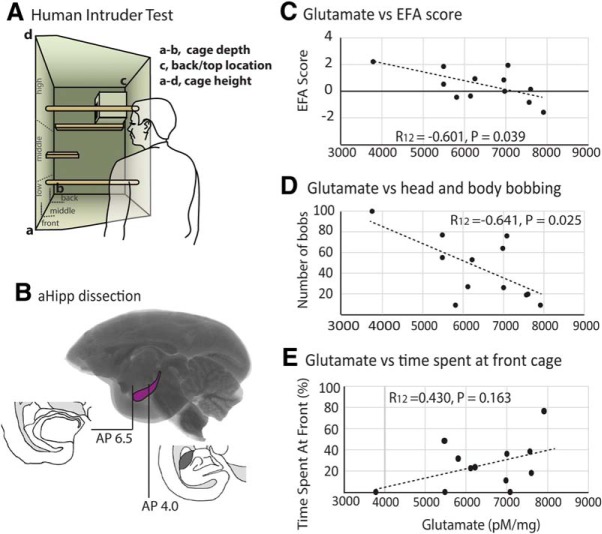
Measures of high-trait anxiety correlate with glutamate levels within the right aHipp. ***A***, Schematic of the human intruder test apparatus detailing the depth (front, middle, back) and height (floor, low, middle, high, top of nest box) locations that the monkey could occupy. ***B***, Schematic showing the dissection of the hippocampus from AP 4 to AP 6.5 before glutamate analysis. Decreased levels of hippocampal glutamate levels correlated with increased EFA score (***C***), increased head and body bobbing (***D***), but not TSAF (***E***).

At ∼2 years of age, high-trait-anxious marmosets were identified according to their performance on a human intruder test, a well-validated test of uncertainty-based anxiety in primates (Carey et al., 1992; Oler et al., 2009; Fox et al., 2010; Mikheenko et al., 2015). In the marmoset version of this test, the monkey is confined to the upper right hand quadrant of their cage away from their cage-mate and a video camera is focused on their quadrant to allow their behavior to be quantified after the test. After an 8 min habituation period, human (wearing an unfamiliar latex human face mask) enters the room, stands 30 cm away from the quadrant front, and maintains eye contact with the monkey for 2 min (Agustín-Pavón et al., 2012) (Figure 1). Because the intent of the unknown human is unclear, as he or she may provide treats or may try and hold/catch the monkey, the uncertainty tends to induce anxious behavior. There are many behaviors that can be analyzed in this paradigm, but one of the most relevant is the location of the animal within the cage. In contrast to normal animals, high-trait-anxious marmosets characteristically retreat from the human intruder to the back (increased average depth from intruder) and/or the top (increased average height from intruder) of the cage and therefore have a high score for time spent at the top/time spent at the back (TSAB) of the cage and a very low score for time spent at the front (TSAF) of the cage. In particular, TSAF has been shown to be sensitive to anxiolytics (Carey et al., 1992; Santangelo et al., 2016) and marmosets with a TSAF score of <9% (based on the lower confidence interval of the median of 63 naive marmosets tested on the human intruder) have been shown to display stable characteristic trait anxious behaviors and blunted serotonin signaling compared with marmosets that scored >25% TSAF (upper 95% confidence interval) (Mikheenko et al., 2015). Therefore, those animals with a TSAF score of <9% were defined as high anxious in this study.

##### Drug treatments.

To investigate whether increasing hippocampal glutamate release altered anxiety behaviors in the human intruder test, five high-trait-anxious animals performed this test on an additional three occasions a minimum of a week apart (four animals had cannulae in area 25, area 32, and the aHipp; two animals had cannulae in the aHipp only). The first and third tests were presented after aHipp saline treatment and the middle test was presented after aHipp LY/CGP treatment. This treatment order allowed us to determine whether any drug effects were being confounded by habituation that might occur upon repeated exposure to a human intruder. To help minimize habituation and to ensure that the intruder was novel in appearance on each occasion, the same intruder wore a different human rubber mask as a disguise for each test. These masks were counterbalanced between animals, covered the whole head and hair of the experimenter, and were close fitting so they did not compromise the ability to maintain eye contact.

### Human intruder analysis

Behavior during the human intruder test was recorded and subsequently scored by an experimenter who was blinded to the drug infusion used. During the 2 min intruder period, the time (in seconds) that the animal spent in particular locations within the cage in terms of height (floor, low, medium, high, top of nest box, or average height) and depth (front, middle, back) was recorded as a percentage of the total time, alongside the number of jumps toward the intruder and the amount of time spent locomoting around the cage (in seconds). Additional measures included species specific behaviors namely, the number of seconds spent head and body bobbing (a marmoset behavior indicative of anxiety; Carey et al., 1992; Agustín-Pavón et al., 2011; Santangelo et al., 2016) and the number of various types of vocalizations based on differences in sound, length, and frequency range. These include the “tsik” call, a proactive alarm call used either singly or repetitively in mobbing situations in response to a potential intruder, “tse and egg” calls, which are both indicative of less proactive alarm/anxiety and are associated with vigilance behavior, and combinations of calls such as “tsik-egg” and “tse-egg,” which may serve to coordinate mobbing, vigilance, and anxiety between group members (Bezerra and Souto, 2008). All of these behavioral variables have previously been shown to contribute to an animal's overall response to a human intruder (Agustín-Pavón et al., 2012) and some have been shown to load onto a single factor that was calculated from an exploratory factor analysis (EFA) that was performed on human intruder test scores obtained as part of a screening procedure on 171 marmosets from the University of Cambridge Marmoset Breeding Colony. This EFA was used to predict the extent to which the different behaviors in the human intruder test are driven by any underlying latent variables. The EFA included: TSAF, TSAB, average height, proportion of time spent in locomotion, number of bobs, egg calls, tsik call, tsik-egg calls, and tse-egg calls. In total, nine factors were identified, but based on the point of inflection on a scree plot, a single factor was extracted accounting for 39.7% of the total variance in behavior (Table 2). The major behaviors that contributed to this factor were the percentage of TSAF, TSAB, the average height of the monkey within the cage, and the number of head and body bobs, tse-egg calls, and locomotion. The minor contributory behaviors were the number of tsik-egg and egg vocalizations, and the influence of the tsik calls was negligible. Specifically, animals that scored highly on this composite score spent more time at the back of the cage and high in the cage, indicating a greater avoidance of the human intruder. They also spent more time head and body bobbing, less time locomoting, and made fewer tsik vocalizations and more egg/tse-egg vocalizations, all signs of anxious behavior in marmosets (Carey et al., 1992; Bezerra and Souto, 2008; Agustín-Pavón et al., 2012; Santangelo et al., 2016). Therefore, the pattern in which the items cluster on this factor are all indicative of anxious behavior and suggest that these different behaviors are acting together to drive one underlying composite behavior that represents an animal's overall anxiety toward the human intruder. This composite anxiety score was therefore calculated for each of the high-anxious animals on each occasion that they performed the human intruder test in this study using *z* scores derived from their performance as a group.

**Table 2.** Factor loadings of individual behaviors within the EFA

Behavior	Proportional loading onto anxiety factor
Average height	0.816
Bobs	0.769
Time spent at back	0.688
Tse egg calls	0.417
Egg calls	0.332
Tsik egg calls	0.323
Tsik calls	−0.091
Locomotion	−0.568
Time spent at front	−0.790

The pattern in which these individual behaviors load onto this EFA factor suggests that it represents the marmosets' anxiety toward the intruder, with a high factor score representing an animal high up and at the back of cage far away from the intruder, remaining relatively still, and performing a lot of head bobs, indicative of high anxiety.

#### Unexpected threat paradigm

Behavioral testing using the unexpected threat paradigm took place within a custom-made, sound-attenuated testing chamber in a dark room. This apparatus enabled the measurement of real-time cardiovascular activity via a receiver situated directly under the animal. Animals were trained to enter a transparent Perspex carry box (l = 21.5, w = 19, h = 25 cm) with air holes and a removable clear Perspex door in which they were transported to the behavioral test apparatus. The Perspex carry box was placed inside the test chamber and the marmoset remained inside of this box at all times during testing. The test chamber was lit by a 3 W bulb (house light) located in the middle of the ceiling of the chamber and contained a computer-controlled speaker and a siren generator (120 dB; Biotronix) through which 75 dB auditory stimuli and a 117 dB siren (mildly aversive loud noise) could be played. The apparatus was controlled by the Whisker control system (Cardinal and Aitken, 2010) and in-house software. Three video cameras were positioned in the test chamber so that the activity of the animal within the Perspex box could be recorded by video software (Power Director; CyberLink) and observed directly by the experimenter during testing.

The aim of this test was to expose marmosets to a particular context with unpredictable threat (aversive loud noise) and then to determine their reactivity to a novel, unfamiliar cue presented within this unpredictable threat context. To investigate the effect of a variety of central brain manipulations on their reactivity to unfamiliar cues, a repeated block design was used involving a 5 d cycle of sessions per block. In each session, the animal was positioned inside the Perspex carry box within the testing apparatus and the house light was on. For the first 4 unpredictable threat days, animals were placed in the familiar environment of the testing box for 25 min and presented intermittently with an aversive sound (unconditioned stimulus, US) and an auditory cue. Each auditory cue was 20 s long, 75 dB, and presented 12 times with an intertrial interval (ITI) of 40–80 s. Each US was a 0.4 s, intrinsically aversive 117 dB siren (Agustín-Pavón et al., 2012) and was also presented 12 times per session with an ITI of 40–80 s. The threat of the aversive US was unpredictable because there was no temporal relationship between the auditory cue and the US, so the best predictor of the aversive noise was the context, namely the test apparatus. On the ambiguous cue probe (day 5), monkeys were presented with a novel, 20 s, 75 dB auditory cue presented 12 times with an ITI of 100–180 s within this same threatening context. No US was presented. All drug manipulations occurred on these ambiguous cue probe days. After the cue probe day, the next cycle then started with the novel auditory cue from the prior ambiguous cue probe day incorporated into the unpredictable threat training.

This paradigm therefore measures the response to an uncertain, novel stimulus in an environment associated with the unpredictable threat of a noise stressor (see Fig. 3*A*). However, because the meaning of the novel cue on the probe session is ambiguous, it is potentially threatening, a potential that is amplified by it being presented within an environment associated with the risk of unpredictable threat. By including an unpaired auditory cue on unpredictable threat sessions, it also becomes part of the context (although to a lesser extent than the testing apparatus) and prevents the marmosets learning that every novel auditory cue presentation (the probe days) is associated with no US presentation. Performing the drug manipulations on the cue probe day therefore allows the investigation of the components of this acute stress response in the absence of the noise stressor itself.

#### Unexpected threat analysis

For cardiovascular analysis, BP data were continuously transmitted by the implanted probe to a receiver (RPC-1; DSI) located beneath the testing apparatus. By comparison with an ambient pressure reference monitor (APR-1; DSI), a calibrated pressure output adapter (R11CPA; DSI) converted the absolute pressure into a gauge pressure in millimeters of mercury. The data were then collected, analyzed, and stored offline using an analog/digital converter (micro 1401; Cambridge Electronic Design) and data acquisition software (Spike2 version 7.01; Cambridge Electronic Design). Any outliers and recording failures in the data were removed (BP values >200 mmHg or <0 mmHg or other abnormal spikes). Data collection was reliable overall, but data gaps of <0.4 s were replaced by cubic spline interpolation and gaps of >0.4 s were treated as missing values. Systolic and diastolic BP events were extracted as local maxima and minima for each cardiac cycle and used to calculate the mean arterial pressure (MAP; diastolic BP + [1/3 (systolic BP − diastolic BP)]) and interbeat intervals (IBIs) used to calculate heart rate (HR) and HR variability (HRV) (the root-mean-square SD measure of the time difference between consecutive IBIs), for the 20 s baseline and 20 s aversive cue periods using custom-written code (RN Cardinal) and the HRV Toolkit (https://physionet.org/tutorials/hrv-toolkit/).

Because HRV is determined by the interval between heartbeats, it reflects how flexible the heart is in responding to internal or external stressors. Decreased HRV is associated with anxiety disorders and depression as well as cardiovascular disease and cardiovascular mortality. HRV reflects the balance between the parasympathetic (i.e., vagal) and sympathetic autonomic cardiac regulation and baroreflex activity and assesses the distinct contributions of vagal and sympathetic activity. Poincaré plots (plots of IBI_j+1_ as a function of IBI_j_) were created and the SD of the points perpendicular to the line of identity (SD1) and the SD of the points along the line of identity (SD2) (Toichi et al., 1997) were then used to derive indices of autonomic activity. There were the cardiac vagal index (CVI), which represents the parasympathetic component of cardiac activity, and the cardiac sympathetic index (CSI), which represents sympathetic activity as well as some parasympathetic and baroreflex activity (Toichi et al., 1997; but see Rahman et al., 2011). For each trial, the mean parameter of interest was calculated for the 20 s aversive cue presentation and for the immediately preceding 20 s baseline (BL) period (the last 20 s of the preceding intertrial interval). Therefore, during each cue presentation, the cue-directed HR was calculated as (HR during the cue) − (HR during BL). HRV was also assessed during each 20 s baseline and cue period. This was possible because the high resting HR of marmosets ensured that an adequate number of IBIs (∼100) were gathered across the two sampling periods (Toichi et al., 1997).

The normal healthy cardiovascular response to an ambiguous cue in a potentially threatening environment would be to show a heightened stress response to the potential threat, an increase in HR and CSI, and a withdrawal of CVI, leading to a transient reduction in HRV in preparation for fight-or-flight (de Geus et al., 1990; Boutcher and Stocker, 1996; Pagani et al., 1997). However, this cardiac responsivity is not seen in high-trait-anxious individuals in response to acute stress, who instead show no differences in cardiovascular function between the stress condition and the baseline. This has been referred to as “cardiovascular blunting” (Ginty and Conklin, 2011; Brindle et al., 2013; Levine et al., 2016; Carroll et al., 2017).

The behavior measured during the unexpected threat paradigm was vigilant scanning (VS), which is defined as a watchful scanning of surroundings accompanied by tense, vigilant body posture (Mikheenko et al., 2010; Agustín-Pavón et al., 2012). The time the animal spent engaged in this behavior during the 20 s cue period and 20 s BL period was scored. Cue-directed VS was calculated as the difference between these two (synonymous to cue-directed HR). Studies of healthy individuals indicate that an acute mild stress response is normally associated with an increases in cue-directed VS as the animal engages with the potential threat (Agustín-Pavón et al., 2012). However, high-trait-anxious individuals show deficits in motivational behaviors and task engagement that could be considered a “behavioral blunting” (Carroll et al., 2017).

A second person blinded to the conditions of the experiment scored a subset of the discrimination sessions. Interscorer reliability was high (*R*_(72)_ = 0.76, *p* < 0.001).

### Experimental design and statistical analysis

Glutamate levels from the 12 monkeys in Experiment 1 were correlated to their anxiety-related behaviors using parametric bivariate correlation analyses in SPSS version 22 (IBM) for both the left and right hippocampi. Human intruder test data from Experiment 2a were analyzed using repeated-measures ANOVA and *post hoc* paired (within-subjects) *t* tests in SPSS using a factor of “test” (saline 1, aHipp LY/CGP, or saline 2). Cardiovascular and behavioral data from the unpredictable threat paradigm (Experiment 2b) were analyzed in R version 3.2.2 (R Core Team, 2016) using repeated-measures ANOVA and *post hoc* paired (within-subjects) *t* tests from the lmerTest package and type III sums of squares with the Satterthwaite approximation for degrees of freedom. Factors included area (levels including aHipp, area 32, area 25, and combinations) and drug (including LY/CGP, saline, and musbac). Although MAP was analyzed throughout, it was highly variable and was not statistically affected by any of the manipulations used in the present study. MAP data are therefore not presented. In all cases *n* = 5 or above gives a high statistical power-to-subject ratio while minimizing the number of animals used.

### Postmortem assessment of cannulae placement

Animals were premedicated with ketamine hydrochloride (Vetalar; 0.05 ml of a 100 mg solution, i.m.; GE Healthcare and Upjohn) before being euthanized with pentobarbitol sodium (Dolethal; 1 ml of a 200 mg/ml solution, i.v.; Merial Animal Health). Animals were then perfused transcardially with 500 ml of 0.1 m PBS, followed by 500 ml of 4% paraformaldehyde fixative solution over ∼15 min. The brain was removed and left in the 4% paraformaldehyde fixative solution overnight before being transferred to 30% sucrose solution for at least 48 h. Brains were then sectioned on a freezing microtome (coronal sections; 60 μm), mounted on slides, and stained with cresyl fast violet. The sections were viewed under a Leitz DMRD microscope (Leica Microsystems). The cannula locations for each animal were schematized onto drawings of standard marmoset brain coronal sections and composite diagrams were then made to illustrate the extent of overlap between animals.

## Results

### Experiment 1

#### Increased trait anxiety is associated with reduced glutamate levels within the primate right aHipp

To assess the contribution of aHipp neurochemistry to the trait-anxious phenotype, glutamate and monoamine levels within the right and left aHipp of marmosets (from Shiba et al., 2014) were compared with the composite anxiety score (as determined by factor analysis) and the individual behaviors displayed in response to an unknown human. Hierarchical linear regression revealed that right aHipp glutamate levels were negatively correlated with the composite anxiety score of the animals, with a higher composite anxiety score correlating with reduced aHipp glutamate (EFA score: *R*_(12)_ = −0.601, *p* = 0.039).

Detailed inspection of correlations between right aHipp glutamate levels and individual performance variables revealed that reduced right aHipp glutamate was associated with the animals moving further away from the human intruder. Therefore, reduced glutamate was associated with an increased amount of TSAB and/or on the top of the nest box, the farthest location possible from the intruder (*R*_(12)_ = −0.649, *p* = 0.022), and a trend toward an increase in average height (calculated from percentage time spent on the floor, low, middle, and high locations within the cage; *R*_(12)_ = 0.533, *p* = 0.075). Reduced aHipp glutamate was also associated with increases in the amount of head and body bobbing, an anxiety-related behavior (*R*_(12)_ = −0.641, *p* = 0.025), but no changes in the time spent at the front of the cage (*R*_(12)_ = −0.430, *p* = 0.163), the amount of locomotion (*R*_(13)_ = 0.384, *p* = 0.217), or the types of vocalizations made in response to the intruder (tse, *R*_(12)_ = 0.209, *p* = 0.514; tse-egg, *R*_(12)_ = 0.515 *p* = 0.087; tsik, *R*_(12)_ = 0.280 *p* = 0.3; tsik-egg, *R*_(12)_ = 0.309 *p* = 0.328; egg, *R*_(12)_ = −0.026 *p* = 0.925).

These relationships were not seen with glutamate levels in the left aHipp because adding left aHipp glutamate to the regression model predicting EFA did not improve the fit (*F*_(1,9)_ < 1, NS). The conclusions were also unchanged by analyzing according to the alternative sequence: left aHipp glutamate did not predict the EFA score (*F*_(1,10)_ < 1, NS) but adding right aHipp glutamate to this model significantly improved the fit (*F*_(1,9)_ = 5.8319, *p* = 0.039). Consistent with this, left aHipp glutamate did not correlate with average height (*R*_(12)_ = −0.0.39 *p* = 0.904), time spent at front (*R*_(12)_ = −0.123, *p* = 0.703), or back/top of nestbox (*R*_(12)_ = −0.241 *p* = 0.450) bobbing (*R*_(12)_ = −0.007 *p* = 0.982), any vocalizations (tse, R_13_ = −0.281, *p* = 0.376; tse-egg, *R*_(13)_ = −0.435, *p* = 0.158; tsik, *R*_(13)_ = 0.161 *p* = 0.616; tsik-egg, *R*_(13)_ = 0.004 *p* = 0.3989; egg, *R*_(13)_ = −0.102 *p* = 0.753) or the amount of the locomotion (*R*_(12)_ = 0.149 *p* = 0.645).

Furthermore levels of DA, 5-HT and NA from both the left and right aHipp did not correlate with any of these individual behaviors or the composite anxiety score. However, both 5-HT and NA from the right aHipp, and DA from the left aHipp showed a positive correlation with the time spent at the back of the cage (5-HT, *R*_(12)_ = 0.678 *p* = 0.015; NA, *R*_(12)_ = 0.656 *p* = 0.021; DA, *R*_(12)_ = 0.678 *p* = 0.015; Table 2). These findings indicate that while overall levels of trait anxiety can be selectively linked to reduction in right aHipp glutamate, other neurochemicals may also contribute to specific avoidance behaviors within this test.

### Experiment 2A

#### Pharmacologically increasing presynaptic glutamate release within the aHipp reverses the high-anxiety phenotype on the human intruder test

**Figure 2.**
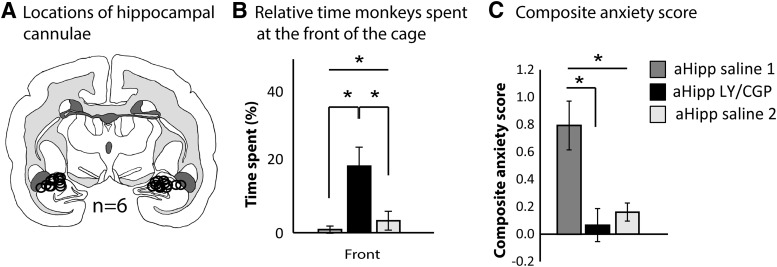
aHipp LY/CGP treatment reversibly increases the TSAF and reduces the EFA score in the human intruder test. ***A***, Schematic showing the position of the cannula tips and cannula locations for each animal. All aHipp cannulae were located within the ranges of AP, 4.8–5.6, respectively, and are plotted here on a single coronal section. Cytoarchitectonic parcellation was based on Burman and Rosa (2009). Circles represent the estimated maximal spread of the muscimol/baclofen or saline infusions (West et al., 2011). ***B***, TSAF is reversibly increased by aHipp LY/CGP infusion, but this is not shown in the composite anxiety score (***C***) due to habituation between the two saline infusions.*p < 0.05.

Pharmacologically boosting aHipp glutamate levels in high-trait-anxious monkeys significantly altered their anxiety-related behavior in response to the human intruder, but did not affect all behaviors equally. Bilateral infusion of aHipp LY/CGP did decrease the composite anxiety score (EFA) indicative of a decrease in anxiety. However, there was also a tendency for EFA scores to show habituation between the first and second saline infusions (saline 1 and saline 2, respectively). Therefore, despite EFA scores correlating with right aHipp glutamate levels in Experiment 1 and showing a decrease after aHipp LY/CGP, its failure to return to saline 1 levels after saline 2 means that habituation effects cannot be ruled out (Fig. 2, Table 3). In contrast, the marked rise in the TSAF after aHipp LY/CGP was not accompanied by habituation between the first and second saline infusions, indicating that the animals experienced less anxiety and spent more time in close proximity to the human intruder after aHipp LY/CGP only. Performance variables of height, movements (jumps, bobs and locomotion), and call types were unaffected.

**Table 3.** Hippocampal levels of 5-HT, dopamine, and noradrenaline do not correlate with any of the behavioral measures sensitive to glutamate in the human intruder test

	Dopamine		Serotonin		Noradrenaline	
aHipp	L	R	L	R	L	R
EFA score	*R*_(12)_ = 0.332, *p* = 0.291	*R*_(12)_ = −0.072, *p* = 0.825	*R*_(12)_ = 0.026, *p* = 0.935	*R*_(12)_ = 0.271, *p* = 0.394	*R*_(12)_ = 0.021, *p* = 0.949	*R*_(12)_ = −0.127, *p* = 0.694
% in depth zones						
Front	*R*_(12)_ = −0.362, *p* = 0.247	*R*_(12)_ = 0.227, *p* = 0.479	*R*_(12)_ = 0.016, *p* = 0.960	*R*_(12)_ = −0.362, *p* = 0.247	*R*_(12)_ = 0.053, *p* = 0.869	*R*_(12)_ = −0.239, *p* = 0.454
Middle	*R*_(12)_ = 0.035, *p* = 0.913	*R*_(12)_ = −0.141, *p* = 0.661	*R*_(12)_ = 0.197, *p* = 0.540	*R*_(12)_ = 0.678, *p* = 0.015	*R*_(12)_ = 0.215, *p* = 0.503	*R*_(12)_ = 0.311, *p* = 0.325
Back	*R*_(12)_ = 0.678, *p* = 0.015*	*R*_(12)_ = 0.176, *p* = 0.585	*R*_(12)_ = 0.030, *p* = 0.927	*R*_(12)_ = 0.678, *p* = 0.015*	*R*_(12)_ = −0.057, *p* = 0.860	*R*_(12)_ = 0.656, *p* = 0.021*
% in height zones						
Floor	*R*_(12)_ = −0.364, *p* = 0.245	*R*_(12)_ = −0.173, *p* = 0.590	*R*_(12)_ = −0.160, *p* = 0.619	*R*_(12)_ = −0.296, *p* = 0.350	*R*_(12)_ = −0.068, *p* = 0.833	*R*_(12)_ = 0.223, *p* = 0.485
Low	*R*_(12)_ = −0.202, *p* = 0.530	*R*_(12)_ = −0.354, *p* = 0.259	*R*_(12)_ = −0.477, *p* = 0.117	*R*_(12)_ = −0.246, *p* = 0.441	*R*_(12)_ = −0.367, *p* = 0.240	*R*_(12)_ = 0.518, *p* = 0.085
Middle	*R*_(12)_ = −0.341, *p* = 0.278	*R*_(12)_ = 0.473, *p* = 0.120	*R*_(12)_ = 0.073, *p* = 0.822	*R*_(12)_ = −0.059, *p* = 0.845	*R*_(12)_ = 0.005, *p* = 0.989	*R*_(12)_ = 0.127, *p* = 0.693
High	*R*_(12)_ = 0.545, *p* = 0.067	*R*_(12)_ = 0.076, *p* = 0.814	*R*_(12)_ = 0.377, *p* = 0.227	*R*_(12)_ = 0.369, *p* = 0.237	*R*_(12)_ = 0.345, *p* = 0.272	*R*_(12)_ = −0.013, *p* = 0.968
Top of nest box	*R*_(12)_ = −0.007, *p* = 0.982	*R*_(12)_ = −0.137, *p* = 0.671	*R*_(12)_ = −0.159, *p* = 0.622	*R*_(12)_ = −0.044, *p* = 0.891	*R*_(12)_ = −0.106, *p* = 0.606	*R*_(12)_ = −0.216, *p* = 0.499
Average height	*R*_(12)_ = 0.328, *p* = 0.298	*R*_(12)_ = 0.014, *p* = 0.966	*R*_(12)_ = 0.091, *p* = 0.779	*R*_(12)_ = 0.220, *p* = 0.493	*R*_(12)_ = 0.032, *p* = 0.922	*R*_(12)_ = −0.281, *p* = 0.376
Movement						
Locomotion	*R*_(12)_ = −0.364, *p* = 0.245	*R*_(12)_ = 0.041, *p* = 0.900	*R*_(12)_ = 0.049, *p* = 0.880	*R*_(12)_ = −0.224, *p* = 0.484	*R*_(12)_ = 0.097, *p* = 0.765	*R*_(12)_ = 0.183, *p* = 0.569
Jumps	*R*_(12)_ = −0.0525, *p* = 0.080	*R*_(12)_ = 0.276, *p* = 0.385	*R*_(12)_ = 0.064, *p* = 0.842	*R*_(12)_ = −0.241, *p* = 0.450	*R*_(12)_ = 0.019, *p* = 0.952	*R*_(12)_ = −0.066, *p* = 0.838
Bobs	*R*_(12)_ = 0.283, *p* = 0.373	*R*_(12)_ = −0.06, *p* = 0.854	*R*_(12)_ = 0.106, *p* = 0.742	*R*_(12)_ = 0.269, *p* = 0.397	*R*_(12)_ = 0.067, *p* = 0.837	*R*_(12)_ = −0.166, *p* = 0.605
Calls						
Tsik	*R*_(12)_ = −0.420, *p* = 0.174	*R*_(12)_ = −0.053, *p* = 0.870	*R*_(12)_ = −5.098, *p* = 0.857	*R*_(12)_ = −0.054, *p* = 0.867	*R*_(12)_ = 0.–114, *p* = 0.725	*R*_(12)_ = 0.197, *p* = 0.540
Tsik-egg	*R*_(12)_ = 0.376, *p* = 0.228	*R*_(12)_ = −0.079, *p* = 0.807	*R*_(12)_ = −0.098, *p* = 0.762	*R*_(12)_ = 0.525, *p* = 0.08	*R*_(12)_ = −0.209, *p* = 0.515	*R*_(12)_ = 0.261, *p* = 0.413
Tse	*R*_(12)_ = −0.143, *p* = 0.657	*R*_(12)_ = 0.211, *p* = 0.510	*R*_(12)_ = −0.238, *p* = 0.438	*R*_(12)_ = −0.101, *p* = 0.755	*R*_(12)_ = −0.265, *p* = 0.406	*R*_(12)_ = −0.029, *p* = 0.928
Tse-egg	*R*_(12)_ = −0.137, *p* = 0.672	*R*_(12)_ = 0.066, *p* = 0.839	*R*_(12)_ = −0.272, *p* = 0.393	*R*_(12)_ = −0.190, *p* = 0.555	*R*_(12)_ = −0.271, *p* = 0.294	*R*_(12)_ = −0.350, *p* = 0.265
Egg	*R*_(12)_ = 0.381, *p* = 0.221	*R*_(12)_ = 0.266, *p* = 0.404	*R*_(12)_ = 0.034, *p* = 0.902	*R*_(12)_ = 0.04, *p* = 0.901	*R*_(12)_ = 0.047, *p* = 0.886	*R*_(12)_ = −0.357, *p* = 0.254

Neither right aHipp 5-HT and NA nor left aHipp DA correlated with the time spent at the back of the cage, suggesting that these neurochemicals may contribute to specific behaviors within the human intruder test.

* *p* < 0.05.

**Table 4.** Summary of human intruder performances after saline and aHipp LY/CGP

	aHipp Saline 1	aHipp LY/CGP	aHipp Saline 2
EFA score*	0.798 ± 0.179	0.06 ± 0.121*	0.161 ± 0.066
% in depth zones			
Front*	0.957 ± 0.957	20.55 ± 4.766*	7.853 ± 4.572*
Middle	40.327 ± 16.098	60.089 ± 10.811	60.704 ± 11.866
Back	32.877 ± 9.965	16.878 ± 7.839	18.990 ± 7.537
% in height zones			
Floor	0.00 ± 0.00	10.136 ± 8.180	10.106 ± 6.613
Low	0.00 ± 0.00	3.7 ± 3.7	4.331 ± 2.842
Middle	2.302 ± 2.302	16.935 ± 7.995	8.904 ± 3.967
High	71.681 ± 8.089	66.738 ± 16.030	64.119 ± 10.841
Top of nest box	19.717 ± 7.8	1.738 ± 1.738	4.414 ± 2.328
Average height	68.758 ± 3.697	58.79 ± 7.503	55.5 ± 5.593
Movement			
Locomotion	2.003 ± 0.832	3.208 ± 0.999	3.623 ± 1.119
Jumps	1.667 ± 0.667	2.5 ± 1.057	3.5 ± 1.057
Bobs	30.333 ± 8.313	19.333 ± 5.714	19.0 ± 7.546
Calls			
Tsik	3.833 ± 3.637	4.0 ± 2.696	1.0 ± 0.516
Tsik-egg	4.667 ± 3.721	4.0 ± 3.235	6.667 ± 5.690
Tse	2.0 ± 1.095	0.667 ± 0.333	0.833 ± 0.477
Tse-egg	6.667 ± 2.951	2.833 ± 2.242	4.00 ± 1.949
Egg	4.823 ± 2.926	1.667 ± 0.333	4.0 ± 1.770

Shown are mean scores for all behaviors analyzed during the human intruder test after each presentation of the test (saline 1, LY/CGP, and saline 2). Data are presented as mean ± SEM.

*p < 0.05. Asterisks in column 1 indicate a main overall effect of the measure; asterisks in columns 2–4 indicate significance compared with the preceding test.

Repeated-measures ANOVA of the composite anxiety score revealed a main effect of test (saline 1, LY/CGP, saline 2) and a significant decrease in anxiety score between saline 1 and aHipp LY/CGP, but no difference between aHipp LY/CGP and saline 2 indicative of habituation (main effect of test, *F*_(2,10)_ = 7.427, *p* = 0.011; first saline vs LY/CGP, *t*_(5)_ = 2.572, *p* = 0.042; second saline vs LY/CGP, *t*_(5)_ = 1.197 *p* = 0.285; Table 3). Repeated-measures analysis of all three depth measures (front, middle, back) also indicated that depth was sensitive to aHipp glutamate levels, but this was not due to alterations in time spent in the middle or back (no main effects of test_3_: middle, *F*_(2,10)_ = 1.98, *p* = 0.189; back, *F*_(2,10)_ = 2.239, *p* = 0.157) and was solely due to a main effect of TSAF. Importantly, this effect was not due to habituation because TSAF was relatively low following both saline infusions, but was markedly increased after aHipp LY/CGP (effect of test_3_, *F*_(2,10)_ = 8.465, *p* = 0.007; first saline vs LY/CGP, *t*_(5)_ = 3.759, *p* = 0.013; second saline vs LY/CGP, *t*_(5)_ = 3.256, *p* = 0.023). No other performance variables of movement, height, or vocalizations were affected (locomotion, *F*_(2,10)_ = 1.17, *p* = 0.348; jumps *F*_(2,10)_ = 1.027, *p* = 0.393; or bobs *F*_(2,10)_ = 2.9, *p* = 0.101; height_5_ × test_3_, *F*_(10,50)_ = 1.69, *p* = 0.108; or vocalizations_6_ × test_3_, *F*_(10,50)_ = 1.776, *p* = 0.09).

Therefore, enhancement of glutamate release in the aHipp specifically ameliorates the high-trait anxiety phenotype by increasing the approach to a human intruder.

### Experiment 2B

#### High-trait-anxious marmosets display low cardiovascular responsivity to the presentation of an ambiguous cue in a threatening environment

**Figure 3.**
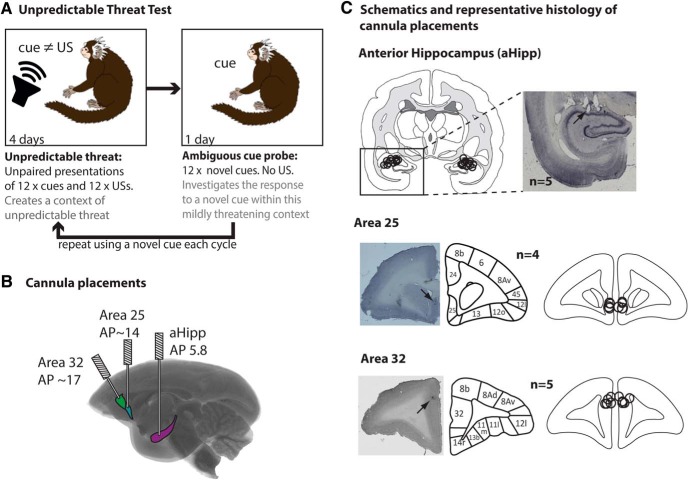
Unpredictable threat test and cannulae schematics. ***A***, In the unpredictable threat test (*n* = 6), animals were placed in a familiar environment for 25 min and played two types of auditory stimuli, a novel auditory cue and a US, for the 4 unpredictable threat days. Each cue was 20 s long, 75 dB, and presented 12 times with an ITI of 40–80 s. Each mildly aversive US was 0.4 s long, 117 dB, and presented 12 times with an ITI of 40–80 s. The threat of the aversive US was unpredictable because there was no relationship between the cue and the US. On the ambiguous cue probe (day 5), monkeys were presented with a novel, ambiguous 20 s, 75 dB cue presented 12 times with an ITI of 100–180 s in the same context. No US was presented. All drug manipulations occurred on ambiguous cue probes. The cycle then repeated with the novel cue incorporated into the unpredictable threat training. ***B***, Glass brain illustrating the rostrocaudal locations of the aHipp, area 25, and area 32 and the intracerebral cannulae targeting each area. ***C***, Representative histological sections with arrows marking the position of the cannula tips and cannula locations for each animal. All aHipp, area 25, and area 32 cannulae were located within the ranges of AP 4.8–5.6, 12.5–14, and 15.8–16.6, respectively, and are plotted here on a single coronal section for each target area. Cytoarchitectonic parcellation was based on Burman and Rosa (2009). Circles represent the estimated maximal spread of the musbac or saline infusions (West et al., 2011).

To assess the contribution of aHipp–mPFC circuitry to anxiety, we first assessed the response of high-trait-anxious animals to a novel auditory cue in a context that has previously been associated with the threat of an unpredictable, aversive loud noise (Mikheenko et al., 2010) (Figure 3*A*).

**Figure 4.**
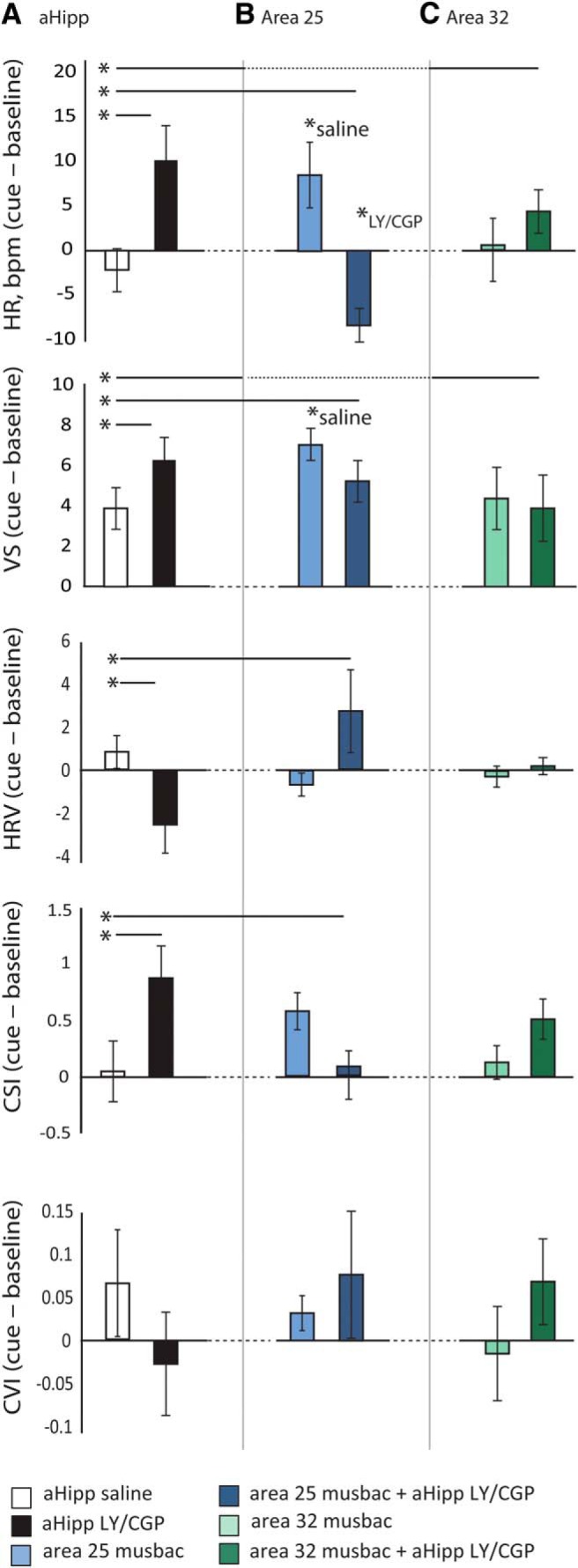
aHipp LY/CGP ameliorates the behavioral and cardiovascular correlates of cue-induced anxiety, but these effects can be blocked by simultaneous area 25 inactivation. Graphs show the changes in HR (beats per minute), VS, HRV, CSI, and CVI under drug and saline conditions during the cue relative to the baseline (the last 20 s of the immediately preceding ITI. Positive numbers indicate an increase from baseline and negative numbers indicate a decrease compared with baseline. Data are shown as mean ± SEM. *p < 0.05. ***A***, Compared with saline, aHipp LY/CGP infusion altered responding in a cue-dependent manner, as assessed by cue-induced increases in HR, VS, and CSI and decreases in HRV. ***B***, Simultaneous aHipp LY/CGP + area 25 inactivation abolished the increases in HR, VS, and CSI that were seen with aHipp LY/CGP alone. Area 25 inactivation also increased HR and VS by itself. ***C***, Simultaneous aHipp LY/CGP + area 32 inactivation did not alter the changes in VS and cardiovascular activity seen with aHipp LY/CGP alone. Area 32 inactivation also had no effect on its own.

**Figure 5.**
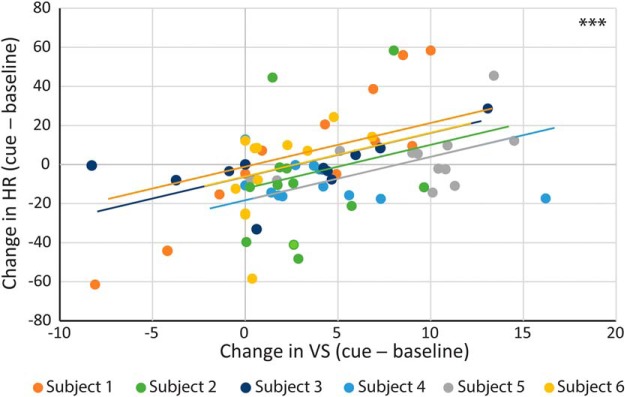
Those animals that showed the smallest cue-induced changes in HR also showed the smallest changes in VS. Within-subjects correlation analysis of the cue-induced changes in HR and VS after infusion of saline into the aHipp revealed that those animals that showed the least HR responsivity also showed the smallest changes in VS. ***p < 0.0001.

Because the cardiovascular responsivity of high-trait-anxious humans is blunted, we first determined whether the cardiovascular and VS response to the novel cue changed compared with the preceding baseline (cue − baseline). High-trait-anxious animals did show a slight increase in VS after bilateral aHipp saline infusion, but like high-trait-anxious humans, they failed to show any cardiovascular alterations because cue-directed HR, HRV, CSI, and CVI were all close to zero (Mikheenko et al., 2010) (one-sample *t* tests compared with no change: VS, *t*_(5)_ = 3.744, *p* = 0.013; HR, *t*_(5)_ = 0.888, *p* = 0.415; HRV *t*_(5)_ = 1.078, *p* = 0.330; CSI, *t*_(5)_ = 0.182, *p* = 0.863; CVI, *t*_(5)_ = 1.043, *p* = 0.345; Fig. 4*A*). Within-subjects correlation analysis of these changes in HR and VS revealed that those animals that showed the least HR responsivity also showed the smallest changes in VS (*F*_(1,65)_ = 19.001, *p* < 0.0001; Fig. 5), indicating a relationship between cardiovascular responsivity and behavioral engagement that we have previously shown to be independent of alterations in arousal or locomotion (Mikheenko et al., 2010; Wallis et al., 2017).

#### aHipp LY/CGP increases cardiovascular responsivity to the presentation of an ambiguous cue in a threatening environment

Compared with the nonresponsiveness of the cardiovascular system to the novel auditory cue after a saline infusion, a marked increase in responsiveness was observed following aHipp LY/CGP infusions. A two-way ANOVA with factors of drug (saline or aHipp LY/CGP) and cue presentations (1–12) revealed that aHipp LY/CGP increased cue-directed HR (main effects of drug_2_, *F*_(1,107.67)_ = 11.25, *p* = 0.001 and drug_2 ×_ cue_12_, *F*_(11,103.22)_ = 2.65, *p* = 0.005, Fig. 4). Similar analyses also revealed an increase in cue-directed CSI (main effects of drug_2_, *F*_(1,108)_ = 4.954, *p* = 0.0281, and drug_2_ × cue_12_, *F*_(11,101.35)_ = 2.63, *p* = 0.005), a decrease in cue-directed HRV (main effect of drug_2_ only, *F*_(1,107.9)_ = 4.956, *p* = 0.028) and no change in CVI (no main effect of drug *F*_(1,107.9)_ = 1.446, *p* = 0.23, drug_2_ × cue_12_, *F* < 1, NS).

In summary, high-trait-anxious animals show a marked lack of cardiovascular responsivity in response to threatening cues, but increasing aHipp presynaptic glutamate release significantly increased both the cardiovascular and behavioral responsivity.

#### Inactivation of area 25 blocks the ability of aHipp LY/CGP to increase cardiovascular responsivity and VS

To determine whether the increases in cardiovascular responsivity induced by aHipp LY/CGP engage aHipp-area 25 circuitry, aHipp LY/CGP infusions were combined with simultaneous inactivation of area 25 with 0.5 μl of musbac.

This simultaneous inactivation was found to reduce the impact of aHipp LY/CGP on HR, VS, HRV, and CSI. Therefore, repeated-measures ANOVA with factors of manipulation (25 musbac, aHipp saline, aHipp LY/CGP, and aHipp LY/CGP + 25 musbac) and cue presentations (1–12) revealed changes in HR, VS, HRV, and CSI, but not CVI (HR, main effect of manipulation_4_ *F*_(3,16.7)_ = 8.98, *p* = 1.49^1 × 10−05^; VS, main effect of manipulation_4_ *F*_(3,177.46)_ = 4.82, *p* = 0.002; HRV, main effect of manipulation_4_ *F*_(3,180)_ = 3.4, *p* = 0.017; CSI, a trending effect of manipulation_4_ *F*_(3,148.22)_ = 2.269, *p* = 0.08, and a manipulation_4_ × cue number_12_ interaction *F*_(33,173)_ = 1.59, *p* = 0.02; CVI, *F*_(3,180)_ = 0.69, *p* = 0.55).

*Post hoc* analysis revealed that simultaneous aHipp LY/CGP + area 25 musbac infusions reversed some of the cue-directed changes that were induced by aHipp LY/CGP alone. Specifically, the increases in HR that were seen after aHipp LY/CGP were abolished to below control levels, with the effects of aHipp LY/CGP + area 25 musbac no longer different from aHipp saline (*t*_(3)_ = 1.8, *p* = 0.157). Similarly, the increases in VS and CSI seen after aHipp LY/CGP were returned to saline levels after aHipp LY/CGP + area 25 musbac (VS, aHipp saline vs aHipp LY/CGP + area 25 musbac *t*_(3)_ = 0.7, *p* = 0.49, aHipp LY/CGP vs aHipp LY/CGP + area 25 musbac *t*_(2)_ = 1.45, *p* = 0.285. CSI, aHipp saline vs aHipp LY/CGP + area 25 musbac *t*_(3)_ = 1.5, *p* = 0.21, aHipp LY/CGP vs aHipp LY/CGP + area 25 musbac *t*_(2)_ = 6.43, *p* = 0.02). Although, numerically, the combined aHipp LY/CGP + area 25 musbac did reverse the reductions in cue-directed HRV induced by aHipp LY/CGP, these did not reach significance due to high variability (aHipp saline vs aHipp LY/CGP + area 25 musbac *t*_(2)_ = 1.5, *p* = 0.27, aHipp LY/CGP vs aHipp LY/CGP + area 25 musbac *t*_(2)_ = 1.2, *p* = 0.27).

For control purposes, we also investigated the effects of area 25 inactivation alone. However, in contrast to the ability of area 25 inactivation to block the enhanced responsivity induced by aHipp LY/CGP, area 25 inactivation on its own also enhanced responsivity in a similar manner to aHipp LY/CGP. Therefore, compared with saline, area 25 inactivation increased measures of both cue-directed HR and VS (HR, *t*_(3)_ = 4.03, *p* = 0.027; VS, *t*_(3)_ = 4.03, *p* = 0.027; Fig. 4*B*). HRV, CSI, and CVI were not affected by area 25 inactivation (*t* < 1, NS). Because these HR and VS findings are very similar to those seen after aHipp LY/CGP, the fact that both measures do not differ from saline after the combined aHipp LY/CGP + area 25 musbac infusions indicates that the combined manipulation not only abolishes the independent effects of aHipp LY/CGP manipulations alone, but also abolishes the effects of 25 inactivation alone. This indicates reciprocal communication between these structures in the behavioral and cardiovascular regulation of high-trait anxiety.

Area 25 activity is therefore an important determinant of aHipp LY/CGP's ability to alter the behavioral and cardiovascular indices of high-trait anxiety in response to an ambiguous cue and highlights the importance of reciprocal aHipp-area 25 connectivity in this phenotype.

#### Inactivation of area 32 does not alter the effects of aHipp LY/CGP

To determine the contribution of aHipp-area 32 circuitry to the effects of aHipp LY/CGP, we also combined aHipp LY/CGP infusions with simultaneous inactivation of area 32. However, unlike area 25, area 32 inactivation did not modulate the effects of aHipp LY/CGP. Repeated-measures ANOVA of the area 32 manipulations with factors of manipulation (32 musbac, aHipp saline, aHipp LY/CGP, and aHipp LY/CGP + 32 musbac) and cue number (1–12) revealed no main effects of manipulation_4_ on cue-directed HRV or CVI (HRV, *F*_(3,180)_ = 1.6, *p* = 0.18; CVI, *F*_(3,179.3)_ = 0.7, *p* = 0.55). Although similar analysis did reveal main effects of manipulation on both cue-directed HR and cue-directed VS (HR, *F*_(3,190.97)_ = 4.1, *p* = 0.007; VS, *F*_(3,189.4)_ = 4.36, *p* = 0.005) and CSI approaching significance (*F*_(3,175.7)_ = 1.6, *p* = 0.055), these effects were not due to a modulatory role of area 32 or to the effects of simultaneous aHipp LY/CGP + area 32 inactivation, but rather to the effects of aHipp LY/CGP alone.

Therefore, although aHipp LY/CGP + area 32 inactivation did slightly reduce the increases in cue-directed HR seen with aHipp LY/CGP alone, this effect was not significant (aHipp LY/CGP vs aHipp LY/CGP + area 32 musbac, *t*_(3)_ = 1.4, *p* = 0.234; Fig. 4*C*). Similarly, aHipp LY/CGP + area 32 musbac did not alter the effect of aHipp LY/CGP on cue-directed VS (*t* < 1, NS, aHipp saline vs area 32 musbac, *t* < 1, NS; aHipp LY/CGP vs aHipp LY/CGP + area 32 musbac, *t*_(3)_ = 1.53, *p* = 0.223), CSI (aHipp saline vs area 32 musbac, *t* <1, NS; or aHipp LY/CGP vs aHipp LY/CGP +area 32 musbac, *t*_(2)_ = 1.89, *p* = 0.199), or CVI (aHipp saline vs area 32 musbac, *t* < 1, NS, aHipp LY/CGP vs aHipp LY/CGP +area 32 musbac, *t* < 1, NS). Control inactivations of area 32 alone revealed no effects on cue-directed HR, VS, CSI (all *t* < 1, NS), HRV, or CVI (*t* > 1, NS). Therefore, activity within area 32 does notaffect the ability of aHipp LY/CGP to increase the behavioral and cardiovascular indices of high-trait anxiety in response to an ambiguous cue.

## Discussion

We have demonstrated that high-trait anxiety in marmosets is correlated with right, but not left, aHipp glutamate levels, with reduced presynaptic glutamate in the right aHipp being associated with increased anxiety. Pharmacologically elevating aHipp presynaptic glutamate release with aHipp LY/CGP in a cohort of high-trait-anxious marmosets reduced their behavioral measures of anxiety in response to an unknown human. Glutamatergic elevations also increased the responsivity of the previously unresponsive cardiovascular systems of these high-trait-anxious animals to unpredictable threat cues and simultaneously increased their vigilant scanning responses to the same cues. Investigation into the specific pathways between the aHipp and mPFC that may underlie these cardiovascular and behavioral effects in high-trait-anxious animals revealed that simultaneous inactivation of area 25, but not area 32, abolished the effects of elevating aHipp glutamate. Therefore, we provide causal evidence in primates to support the hypothesis that aHipp glutamatergic hypofunction contributes to specific aspects of the behavioral and autonomic correlates of high-trait anxiety via an aHipp–area 25 pathway.

High-trait-anxious marmosets in the current study showed a marked lack of cardiovascular responsivity to a threatening cue and similarly low levels of VS. These findings contrast with the increases in HR and/or BP and VS that normally signal increased fear in nonanxious animals during aversive fear conditioning and in high-anxious marmosets responding in a similar, but less aversive, unpredictable threat paradigm (Mikheenko et al., 2010; Shiba et al., 2014; Wallis et al., 2017). Although one interpretation of these findings is that the paradigm was not aversive enough to cause a fear response, this is unlikely given the known aversive impact of the noise cue used (Wallis et al., 2017). A more likely interpretation is based on the findings in high-trait-anxious or depressed humans, who show blunted cardiovascular responsivity to such stressors and reduced behavioral engagement that is most extreme in those individuals who show the greatest cardiovascular blunting (Ginty et al., 2015). Therefore, the lack of cardiovascular responsivity to the threatening cues in the high-trait-anxious marmosets may be equivalent to the cardiovascular blunting seen in high-trait-anxious humans, although for confirmation, a direct comparison with low-anxious animals is required. Certainly, however, we have reported previously a reduction in BP, reminiscent of the blunted response seen here, in a subset of high-anxious marmosets following repeated exposure to aversive Pavlovian-conditioned stimuli (Shiba et al., 2014), an effect not seen in low-anxious marmosets that displayed the expected elevation in cardiovascular activity to an aversive Pavlovian-conditioned stimulus. Furthermore, like humans, the high-trait-anxious marmosets with the smallest HR changes also showed the smallest VS changes in Experiment 2, so the low levels of VS seen in the present study may reflect decreased behavioral engagement with the cue. These findings therefore most likely indicate that the high-trait-anxious monkeys display a similar blunted phenotype to high-trait-anxious humans and that this phenotype is linked to reduced hippocampal glutamate.

Hippocampal glutamatergic hypofunction has previously been linked to affective illness severity (de Diego-Adeliño et al., 2013). However, despite strong evidence that a hippocampal network mediates stressor-evoked behavioral and cardiovascular activity, few studies have investigated the role of the hippocampus in cardiovascular responsivity (Neafsey et al., 1993; Bär et al., 2016; Kuntze et al., 2016; Ajayi et al., 2018). Here, we show that increasing aHipp glutamate with aHipp LY/CGP causes corresponding increases in the HR and VS response to a threatening cue, along with increases in CSI and decreased HRV. These are the components of the fight-or-flight response that are normally shown in nonanxious animals responding to threat and suggest that aHipp LY/CGP has had an anxiolytic effect by normalizing the previously blunted responses. This is supported by the anxiolytic effects of aHipp LY/CGP in the human intruder test. Here, aHipp LY/CGP increased the time animals spent at the front of the cage and reduced the time spent deep in the cage, consistent with an increased approach to, and engagement with, the potentially threatening intruder. Therefore, both the increased VS in the unexpected threat paradigm and the increased approach behavior in the human intruder test may reflect increased engagement with the potential threat (be it aversive cue or human intruder). Together, these findings indicate that elevation of aHipp glutamate can ameliorate the high-trait-anxiety phenotype, in part by returning cardiovascular and behavioral reactivity to the healthy, nonblunted state (via a paradoxical increase in the fight-or-flight response). Glutamate within the aHipp is clearly an important regulator of the cardiovascular and behavioral correlates of high-trait anxiety.

Given that our data associated elevated trait anxiety with reductions of right aHipp glutamate only, we could speculate that these alterations are due to the effect of increasing glutamate within the right and not the left aHipp despite our use of bilateral aHipp LY/CGP infusions. Our manipulation data and human neuroimaging data are inconclusive regarding the laterality of the hippocampal abnormalities that are apparent in the affective disorders (Malykhin and Coupland, 2015) and no hemispheric differences were seen in the hippocampal response to early life stress (maternal deprivation) in marmosets (Law et al., 2009). However, some neuroimaging studies do report a stronger relationship between right aHipp neurochemistry and the affective disorders compared with the left hippocampus (de Diego-Adeliño et al., 2013). Furthermore, brief, repeated exposure to a novel environment during the early life of rodents has been shown to preferentially alter the synaptic plasticity and volume of the right, not left, hippocampus (Verstynen et al., 2001; Tang et al., 2008). These rodent changes were small (and equivalent changes may have been masked by the lower group sizes in the marmoset study), but nevertheless suggest the right hippocampus may be particularly important for translating the effects of early life experiences into adult temperament.

The hippocampus is connected to many other limbic brain regions through which it can enact these effects. These regions include the amygdala and the mPFC, which have been extensively studied in rodent models of anxiety and fear behavior, but less so with respect to cardiovascular regulation. Certainly, rodent studies of threat regulation emphasize a tripartite mPFC–vHipp–amygdala circuit (Sierra-Mercado et al., 2011; Padilla-Coreano et al., 2016) and stronger amygdala–hippocampal network activity has been associated with higher levels of human trait anxiety (Kirkby et al., 2018). Although we have not investigated the contribution of the amygdala in this study, we have previously shown that levels of 5-HT are reduced in the amygdala of high-anxious marmosets (Mikheenko et al., 2015), and the aHipp sends double-projecting neurons to both the mPFC and the basal amygdala through which both regions can be modulated simultaneously (Kim and Cho, 2017).

Communication specifically between the vHipp and the mPFC is vital for the behavioral correlates of threat conditioning in rodents, and studies have primarily emphasized the role of vHipp connectivity with the prelimbic (PL), rather than the infralimbic (IL), subregion (LeDoux, 2000; Adhikari et al., 2010; Sierra-Mercado et al., 2011; Sotres-Bayon et al., 2012; Padilla-Coreano et al., 2016). Because anatomical and connectivity studies suggest that rodent IL and PL are homologous to primate areas 25 and 32, respectively (Vertes, 2006; Vogt and Paxinos, 2014; Heilbronner et al., 2016), these rodent studies predict the importance of primate–area 32 connectivity for threat regulation. Despite this, the current data highlight the importance of aHipp–area 25 connectivity for the regulation of the behavioral and cardiovascular indices of cue-directed high-trait anxiety and provide causal support for human neuroimaging studies highlighting the importance of aHipp-area 25 connectivity in the regulation of negative emotional behavior (Dickie et al., 2011; Hamilton et al., 2011; Treadway et al., 2015). One possible explanation for these differences is that hippocampal glutamate differentially regulates these mPFC subregions in healthy and high-trait-anxious states. This would also explain why v/aHipp lesions and glutamate antagonism in nonanxious rodents and primates are anxiolytic (Chudasama et al., 2008, 2009; Barkus et al., 2010), yet increasing v/aHipp glutamate in high-trait-anxious rats and marmosets is also anxiolytic (Marrocco et al., 2012; present study). However, confirmation of this hypothesis requires the testing of nonanxious animals on the current paradigm for comparison with the high-anxious cohort. Alternatively, the rodent IL and PL may have different functions from primate areas 25 and 32. Recent behavioral findings in marmosets showed that inactivation of areas 25 and 32 had opposite autonomic and behavioral effects on fear discrimination and extinction to these putative rodent homologs, indicating uncertainty over the functional analogy of IL/25 and PL/32 (Wallis et al., 2017). Inactivation of the IL also has no effect on cardiovascular control during baseline or stressful conditions, whereas area 25 inactivation causes significant bradycardia (Müller-Ribeiro et al., 2012; Wallis et al., 2017). Nevertheless, vHipp–IL circuitry in the rat has been implicated in cardiovascular regulation because electrical stimulation of the vHipp in anesthetized rodents is only able to elicit a depression of cardiovascular activity (decreased HR) if the IL is intact (Ruit and Neafsey, 1990; Gianaros et al., 2004). However, it should be noted that, in both species, the hippocampus appears to connect primarily with the mPFC regions in which inactivation reduces fear.

Finally, inactivations of areas 25 and 32 alone had distinct effects. Inactivation of area 25 caused similar increases in HR and VS to aHipp LY/CGP, which suggests that area 25 inactivation is also anxiolytic in this paradigm. This replicates previous findings in which area 25 inactivation reduced the behavioral and cardiovascular indices of cued fear in healthy marmosets (Wallis et al., 2017; but see Gianaros et al., 2004; Critchley et al., 2011). The findings that area 25 inactivation and aHipp LY/CGP have the same effects independently, but their simultaneous infusion blocks these effects, suggests functional dependence of these two structures in the regulation of high-trait anxiety. In contrast, area 32 inactivation had no effect on the cardiovascular and VS responses induced by the novel cue, and the combined aHipp LY/CGP + area 32 inactivation also failed to modulate the anxiolytic effects of aHipp LY/CGP alone. This diverges from the increase in threat responses seen after area 32 inactivation (with GABA agonists) during cued fear conditioning in nonanxious marmosets (Wallis et al., 2017) and the positive relationship between perigenual GABA levels and human trait anxiety (Delli Pizzi et al., 2016). It is also different from the increase in HR and foreboding, or negative bias, that is seen after perigenual ACC stimulation in nonanxious epileptic patients and macaques, respectively, which may have preferentially activated GABAergic interneurons (Amemori and Graybiel, 2012; Parvizi et al., 2013). However, it may not be possible to detect further increases in defensive behaviors in response to certain or uncertain threat in high-trait-anxious animals, which may already be displaying maximal levels of this behavior. Clearly, the differing contributions of areas 25 and 32 to the modulation of threat responses has implications when considering treatment, particularly when considering GABAergic medications such as benzodiazepines (Bruijn et al., 2001).

To conclude, we have shown that aHipp glutamatergic hypofunction is a key predictor of a high-trait-anxiety phenotype in marmoset monkeys and that, by pharmacologically normalizing such hypofunction, we can also normalize the abnormal behavioral and cardiovascular blunting associated with this phenotype. We provide evidence that an aHipp–area 25 circuit, but not an aHipp–area 32 circuit underlies this specific anxiolytic effect and highlight the aHipp–area 25 circuit as a potential therapeutic target. Together, these findings demonstrate the importance of investigating both the behavioral and autonomic aspects of emotional responding and suggest that investigating cardiovascular function in rodents may be a valuable adjunct when comparing rodent and primate mPFC function.
